# Midline-1 inhibited high glucose-induced epithelial-mesenchymal transition, fibrosis and inflammation through WNT/β-catenin signaling in benign prostatic hyperplasia

**DOI:** 10.3389/fendo.2025.1543295

**Published:** 2025-03-26

**Authors:** Xun Fu, Hao Zhang, Jiang Liu, Yan Li, Zhen Wang, Shu Yang, Daoquan Liu, Yongying Zhou, Ping Chen, Michael E. DiSanto, Hongjun Li, Xinhua Zhang

**Affiliations:** ^1^ Department of Urology, Zhongnan Hospital of Wuhan University, Wuhan, China; ^2^ Department of Urology, Peking Union Medical Collage Hospital, Beijing, China; ^3^ Department of Surgery and Biomedical Sciences, Cooper Medical School of Rowan University, Camden, NJ, United States

**Keywords:** benign prostatic hyperplasia, epithelial-mesenchymal transition, fibrosis, high glucose, inflammation, MID1

## Abstract

**Background and objects:**

Benign prostatic hyperplasia (BPH) is a common disease that impairs the life quality of elderly men. The close relationship of BPH and diabetes has been generally established, however, the exact molecular mechanism remains unclear. Midline-1 (MID1) is an E3 ubiquitin ligase belonging to Tripartite Motif family and its involvement in the initiation and progression of many diseases, such as diabetic kidney disease has been well accepted. This study aims to illuminate the potential impact of high glucose (HG) on prostatic cells and elucidate the molecular role of MID1 in the development of BPH.

**Methods:**

In this work, human prostate specimens and cultured human prostate cell lines (BPH-1 and WPMY-1) were employed. The impact of HG treatment on these two lines was assessed and the expression and localization of MID1, along with its potential downstream target protein phosphatase 2A (PP2A), were determined using multiple experimental methods. MID1-overexpressing cell models were further used to investigate the function of MID1 in regulating inflammation, fibrosis and epithelial-mesenchymal transition (EMT).

**Results:**

Herein we demonstrate diabetic individuals with BPH had lower expression of MID1 and higher expression of the catalytic subunit of PP2A (PP2Ac), larger prostate volume, higher international prostate symptom score (IPSS) and lower Qmax than non-diabetic groups. On a cellular level, HG treatment inhibited the expression of MID1, thus stimulating cellular proliferation and triggering EMT, fibrosis and inflammation of two prostatic cells via enhanced WNT/β-catenin signaling.

**Conclusions:**

In general, our novel data demonstrate targeting MID1 might be a promising area of medical treatment for patients with both BPH and diabetes.

## 1 Introduction

Benign prostatic hyperplasia (BPH) is a frequent condition in aging men with adverse effect on men’s health (1). The prevalence of BPH increases with age (2), reaching 50-60% in men aged 60-69 and 80-90% in men aged >80 (3). Novel insights into the pathogenic mechanism of BPH have uncovered several possible causative factors of this disease – sex hormones (androgen and estrogen), the imbalance of androgen-estrogen ratio, the dysregulation of cell proliferation and apoptosis, the interaction between stromal and epithelial cells, inflammation, and growth factors (2, 4, 5). However, the exact pathogenesis of BPH still remains unclear.

Diabetes mellitus (DM) is a systemic disease and characterized by glucose metabolism disorders. Metabolic syndrome including DM has been reported to increase the risk of BPH and prostate volume (6). A descriptive study showed that the fasting blood glucose level positively correlated with prostate volume and international prostate symptom score (IPSS), and the progression of BPH can be prevented by lowering the blood glucose levels (7). Consistent with Zhou’s results, Ozcan et al. observed larger prostate, higher IPSS scores and more post-void residual urine among diabetic patients with BPH compared to non-diabetic individuals (8). However, it is worth noting that the majority of present data are observational studies, and little is known regarding the molecular connection between these two clinical entities. A newly-published research illuminated that high glucose levels stimulated proliferation, activated epithelial-mesenchymal transition (EMT), and inhibited apoptosis of prostatic cell lines (BPH-1 and RWPE-1) by downregulating PDK4, providing evidence for the potential of PDK4 as a therapeutic target for BPH (9). Indeed mechanistically, high levels of glucose (HG) could trigger EMT in prostatic cells (9, 10), while prostatic hyperplasia has been attributed to the accumulation of mesenchymal-like cells derived from the epithelium and endothelium of prostate gland, rather than the proliferation of prostatic stromal cells (11). Apart from EMT event, HG is known to promote the generation of reactive oxygen species (ROS), excessive production of which triggers inflammation and fibrosis of tissue (12). Inflammation is one of the causes of prostate enlargement and prostatic fibrosis, and both inflammation and fibrosis are able to result in increased rigidity of the prostate (13, 14). Consequently, it appears that prostate inflammation, fibrosis and EMT are three critical events participating in the initiation of BPH among diabetic patients.

Midline-1 (MID1) is an E3 ubiquitin ligase belonging to the Tripartite Motif (TRIM) family and it is also known as TRIM18 (15). MID1 could promote the degradation of the catalytic subunit of protein phosphatase 2A (PP2Ac, a critical cellular regulator) (16), by which MID1 exerts its function in the origin and development of inflammation, fibrosis, EMT (17, 18). On the other hand, high glucose levels have been shown to affect the expression of MID1 (18). Taken together, MID1 might be the molecular basis of the initiation of BPH among diabetic patients.

In this study, we investigated the contribution of HG to the initiation and progression of BPH. We also tried to unravel the role of MID1 in inhibiting HG-induced EMT, fibrosis and inflammation of prostate cells and its underlying mechanism, in order to establish another molecular connection between diabetes and BPH.

## 2 Materials and methods

### 2.1 Human tissues and clinical data

Normal and hyperplastic human prostatic tissues used in the study were obtained from Zhongnan Hospital of Wuhan University. Prostatic samples confirmed to be either normal or benign hyperplastic by two separate pathologists met the inclusion criteria. Any tissues either pathologically identified as prostate cancer or from patients with metabolic disorders such as hypertension, obesity and hyperlipidemia were excluded. Among a total of 135 included prostate specimens, 17 normal samples were donated by brain-dead men (mean age: 29.6 ± 4.8 years old) and 118 hyperplastic ones were obtained from patients with BPH (mean age: 70.1 ± 6.3 years old) who underwent transurethral prostate resection in the department of urology, Zhongnan Hospital of Wuhan University. The written informed consent from Zhongnan Hospital of Wuhan University is available and our protocol was approved by the Hospital Medical Ethics Committees. Each prostate sample was segmented into two strips: one for qRT-PCR and Western blot analysis (stored in liquid nitrogen), and one for immunofluorescence microscopy and tissue microarray (TMA) construction (stored in 10% neutral buffered formalin).

In addition, clinical data of all such patients were collected, including age, body mass index (BMI), fasting blood glucose (FBG), HbA1c, prostate volume, total prostate specific antigen (tPSA), free prostate specific antigen (fPSA), IPSS scores and maximum urine flow rate.

### 2.2 Cell culture

Human benign prostatic hyperplasia epithelial cell line (BPH-1) (Cat. #BNCC339850) was purchased from Procell Co., Ltd., Wuhan, China. This line was cultured in RPMI-1640 medium (Gibco, China) with 10% fetal bovine serum (FBS, GIBCO, Australia). SV40 large T antigen immortalized stromal cell line (WPMY-1) (Cat. #GNHu36) was purchased from Shanghai Institute of stem cells, Chinese Academy of Sciences, and cultured in DMEM medium (Gibco, China) with 5% FBS. Both were cultured at 37°C, under 5% CO_2_ conditions in the cell incubator.

### 2.3 Cell transfection

MID1 pcDNA3.1-3×Flag-C plasmid (pcDNA3.1-MID1) and empty vector were designed by Fenghuishengwu Co., Ltd. in Changsha, China. A total of 2μg of either pcDNA3.1-MID1 or empty vector was diluted in Opti-MEM reduced serum medium and mixed with Lipofectamine^®^2000 (Invitrogen, USA) following manufacturers’ instructions. The complexes were then added to 6-well plates that BPH-1 and WPMY-1 cell lines had been previously seeded in. After transfection for 6 ~ 8 h, the mixture was removed and cells were cultured for 48h and then harvested. Transfection efficiency of pcDNA3.1-MID1 used in the experiment was confirmed at transcriptional and translational levels using qRT-PCR and western blot analysis, respectively.

### 2.4 β-catenin inhibitor treatment for rescue experiments

To inactivate WNT/β-catenin signaling, a β-catenin inhibitor named ICG-001 (MedChemExpress, China) was used in our study. Two prostate cell lines (BPH-1 and WPMY-1) were treated with ICG-001 (dissolved in DMSO beforehand) at six different concentrations (0 ~ 6 μM) so as to determine the experimental dose of ICG-001 (total amount of DMSO remains the same between different groups). In the following experiments, prostatic cells were treated with ICG-001 at 4 μM (the optimal dose) after either pcDNA3.1-MID1 or empty vector transfection. Controls were treated with the same amount of 0.1% DMSO. After culturing for 48 h, cells in 6-well plates were harvested for following CCK8, qPCR and western blot experiments.

### 2.5 Cell Counting Kit-8 assay

Two prostate cell lines (BPH-1 and WPMY-1) were harvested after transfection for 6-8 h. Next, those collected cells were seeded in 96-well plates and then incubated in the cell incubator for 0, 24, 48 or 72h respectively. At different points, a total of 10 μL CCK-8 solution (Sangon Biotech, Shanghai, China) was added to each well and those cells were incubated in the dark for another one hour. The absorbance of each well at 450 nm was determined using a microplate reader (ThermoLabsystems, Vantaa, Finland).

### 2.6 Total RNA extraction, reverse transcription and quantitative real time PCR

Total RNA was extracted from either collected prostate tissues or cells with RaPure Total RNA Micro Kit (Magen, China) and Trizol reagent (Invitrog, Carlsbad, CA, USA) based on the manufactures’ protocol. The concentration and purity of extracted RNA was determined using NanoPhotometer spectrophotometer (IMPLEN, Westlake Village, CA, USA). A total of 1 μg RNA was reverse-transcribed to cDNA with the ABScript II RT Master Mix (ABclonal, Wuhan, China) following the manufacturers’ instructions. Gene amplification was performed by qRT-PCR using a Bio-Rad CFX96 system (Hercules, CA, USA) and sequences of all primers employed are listed in **Supplementary Table S1**. At least three different RNA samples from different prostate tissues were used to accurately determine mRNA expression of each gene. Relative mRNA content was calculated using the 2^−ΔΔ^ *CT* method.

### 2.7 Western blot analysis

Total protein was isolated from either prostate tissues or cells in Radioimmunoassay Buffer (Shanghai Beyotime Biotechnology Co., Ltd., Shanghai, China). A total of 20 μg protein samples were electrophoretically separated on 10% sodium dodecylsulfate-polyacrylamide (SDS-PAGE) gels (Wuhan Boster Biological Technology Ltd., Wuhan, China) at 80 V and then transferred to polyvinylidene fluoride (PVDF) membrane (Millipore, Billerica, MA, USA) for 90 min at 274 mA. Next, the membrane was blocked in 5% nonfat dried milk for 2h. After washing in PBS for three times, the PVDF membrane was successively incubated in primary antibody (**Supplementary Table S2**) overnight at 4 °C and secondary antibody: goat anti-rabbit IgG or goat anti-mouse IgG (**Supplementary Table S3**) for 2h at room temperature. After washing, the bands were detected via an enhanced chemiluminescence kit (Thermo Scientific Fisher, Waltham, MA, USA) on a Tanon-5200 ECL imager (Tanon, Shanghai, China). All bands were densitometrically quantified using Image J software.

### 2.8 Immunofluorescence staining of prostate cells

The coverslip that prostate cells grow on in 6-well plate was first washed by PBS and successively fixed with 4% paraformaldehyde solution (PFA) for 30 min and permeabilized with 0.1% Triton X-100 at room temperature for 5 min. After blocking in 10% BSA at 37°C for 1 h, the coverslip was successively incubated with primary antibody (**Supplementary Table S2**) at 4°C overnight and Cy3 or FITC labeled secondary antibody (**Supplementary Table S3**) in humidified air at 37°C for 1 h. The nucleus was labeled blue with 2 μg/mL 4′,6-diamidino-2-phenylindole (DAPI). Images were analyzed with a Laser Scanning Confocal fluorescence microscope (cat. no. IX73; Olympus, Japan).

### 2.9 Immunofluorescence staining of tissue samples

Prostate specimens were fixed with 4% PFA overnight at 4°C prior to paraffin embedding and sectioning with a special tissue processor (Thermo Fisher Scientific, Cat. #STP120) and rotary microtome (Thermo Fisher Scientific, Cat. #HM325). Paraffin sections (4 μm) were incubated with MID1 primary antibody (**Supplementary Table S2**) and secondary antibody (**Supplementary Table S3**) in turn. The nucleus was labeled blue with 2 μg/mL DAPI. All images were analyzed with above-mentioned Confocal fluorescence microscope.

### 2.10 Immunohistochemistry and clinical correlation analysis

The paraffin sections of prostatic tissues were obtained from our constructed TMA including normal and hyperplastic specimens. In construction of TMA, representative specimens from prepared paraffin-embedded tissues were used, i.e. a total of 135 cores (17 normal and 118 benign hyperplastic, 1.5 mm for each). The tissue paraffin with constructed TMA was continuously sliced into 4‐μm‐thick sections. Such tissue sections were successively incubated with MID1 and PP2Ac primary antibody respectively (**Supplementary Table S2**), and with secondary antibody (**Supplementary Table S3**). Stained sections were imaged using Olympus-DP72 light microscope (Olympus, Japan). Positive area in each image represented relative expression of gene of interest within prostate gland, i.e. intraprostatic levels of MID1 and PP2Ac. In analysis for clinical information, Pearson correlation coefficient and Spearman’s rank correlation coefficient were calculated and utilized to depict correlation between MID1, PP2Ac and several clinical parameters of BPH. Many data-fitting methods, including linear regression and Loess regression, were tried and used in order to visually validate our correlation assumptions.

### 2.11 Statistical analysis

All experiments in our study were performed at least three times. All data were shown in the form of mean ± standard deviation (SD). GraphPad Prism v 5.01 and SPSS v 25.0 were applied for Student’s t-test and one-way ANOVA. *p* < 0.05 was considered to be statistically significant.

## 3 Results

### 3.1 Clinical parameters differ among diabetic and non-diabetic patients with BPH

Trying to identify the association of glucose level and BPH, we first collected the clinical information of 104 patients with BPH from Zhongnan Hospital. Our chart demonstrated that patients with concomitant DM had higher FBG and HbA1c levels (*p*<0.001), a larger prostate gland (*p*<0.001) and higher IPSS scores (*p*<0.05) than those without (age and BMI showed no significant differences between the two patient groups) (**Table 1**). The maximum flow rate (Q_max_) was also significantly lower in the group with DM (*p*<0.05) (**Table 1**). No statistically significant differences were observed between the two groups in terms of other clinical parameters.

**Table 1:** Clinical parameters of diabetic and non-diabetic patients with BPH.

Clinical parameters	Patients with BPH (n=30)	Patients with BPH and DM (n=30)	*p* value
Age	69.4 ± 7.8	70.1 ± 7.4	0.996
BMI (kg/m^2^)	23.9 ± 2.9	23.3 ± 2.6	0.530
FBG (mmol/L)	5.2 ± 0.6	9.0 ± 1.3	<0.001
HbA1c (%)	5.4 ± 0.5	7.1 ± 0.9	<0.001
PV (mL)	46.6 ± 18.3	86.5 ± 32.4	<0.001
tPSA (ng/mL)	8.1 ± 8.9	6.3 ± 5.2	0.603
fPSA (ng/mL)	1.8 ± 2.0	1.2 ± 0.7	0.413
IPSS	21.0 ± 3.9	26.8 ± 5.6	<0.05
Qmax (mL/s)	16.3 ± 9.6	8.3 ± 3.8	<0.05

BMI, body mass index; FBG, fasting blood glucose; PV, prostate volume; tPSA, total prostate specific antigen; fPSA, free prostate specific antigen; IPSS, international prostate symptom score; Qmax, maximum urine flow rate.

### 3.2 High glucose treatment stimulates proliferation of prostatic cells and activates EMT, fibrosis and inflammation process

In this study, BPH-1 (epithelial cell lines) and WPMY-1 (stromal cell lines) were employed to investigate the impact of high glucose treatment and genetic changes on prostatic epithelium and stroma, respectively. We treated BPH-1 and WPMY-1 cell lines with six different concentrations of glucose: 0, 12.5 mM, 25 mM, 37.5 mM, 50 mM and 62.5 mM, to explore the pharmacological effect of glucose treatment at different doses on the two lines. After treatment for 48h, the proliferative rate of both cell lines was evaluated by CCK8 assay. Data from the two bar charts illustrated that HG treatment enhanced proliferative rates of BPH-1 and WPMY-1 cell lines in a dose-dependent manner. In comparison with no glucose addition (0 mM), 12.5 mM glucose had no perceptible impact on prostate cell proliferation (*p*>0.05), while 25 mM glucose significantly affected the two cell lines (*p*<0.05), with 37.5 mM, 50 mM and 62.5 mM glucose inducing a more significant effect (*p*<0.001) (**Supplementary Figure S1**). Meanwhile, there was no statistically significant difference between the influences of 50 mM and 62.5 mM glucose treatment on prostatic cell proliferation (**Supplementary Figure S1**). Thus, we finally chose three different concentrations of glucose: 0, 25 mM and 50 mM in this study to determine the potential impact of high glucose treatment on BPH-1 and WPMY-1 cell lines.

**Figure 1:**
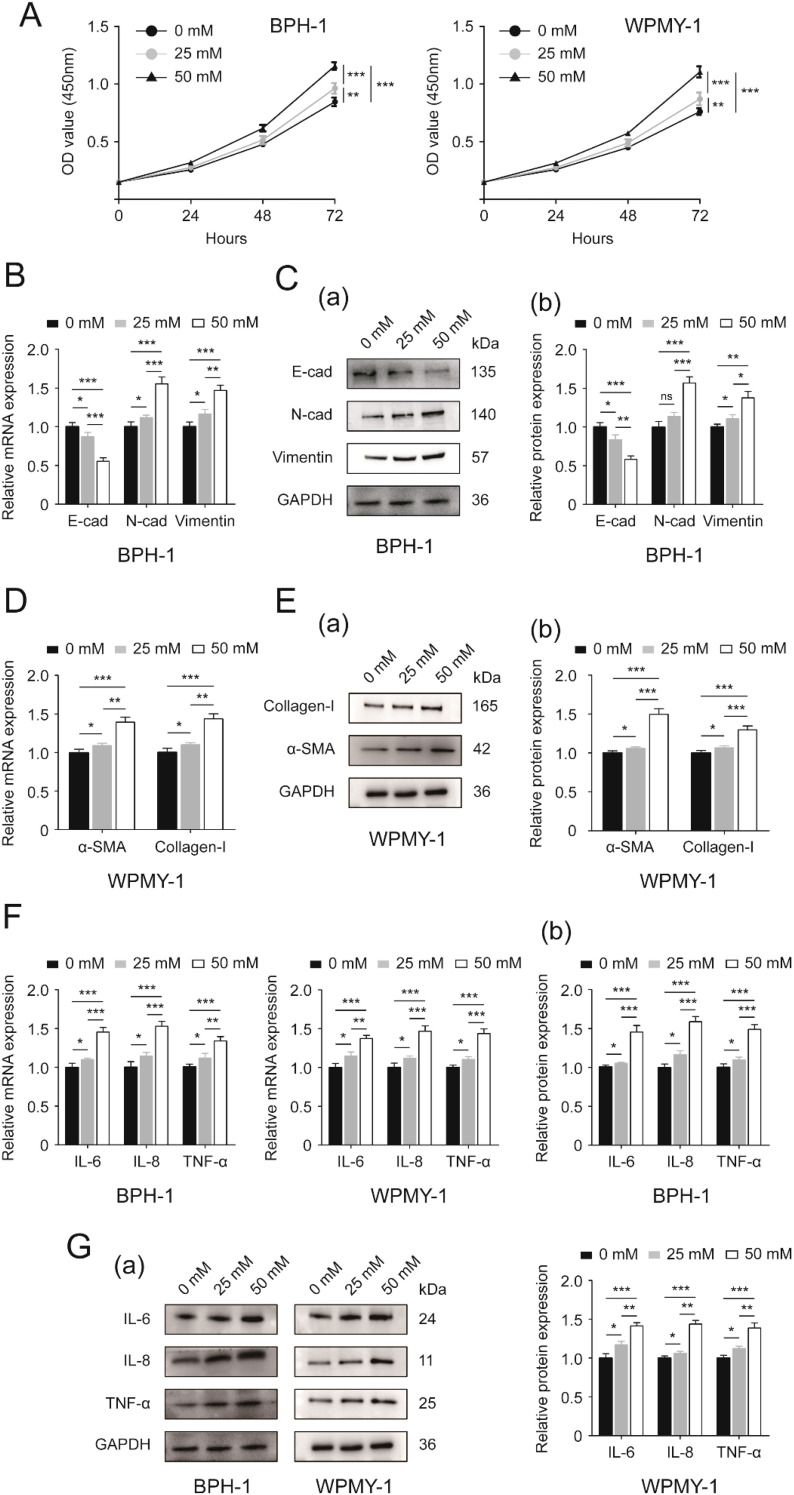
Effects of increasing glucose concentration on two prostatic cell lines. **(A)** Proliferative rates [OD value (450mm)] of BPH-1 and WPMY-1 cells at different time points (0, 24, 48 and 72h) following 0, 25 and 50 mM glucose treatment determined by CCK8 assay. **(B)** The expression of EMT markers (E-cad, N-cad and vimentin) at the transcriptional level in BPH-1 cell line upon 0, 25 and 50 mM glucose treatment. **(C)** Immunoblot assay (a) and relative densitometric quantification (b) for three EMT molecular markers in BPH-1 cells under control (0) and experimental (25 and 50 mM) glucose conditions. **(D)** The mRNA expression of fibrosis biomarkers (α-SMA and collagen-I) in glucose-treated WPMY-1 cell line (0, 25 and 50 mM). **(E)** The expression of α-SMA and collagen-I at the protein level in WPMY cells upon control (-) and experimental (25 and 50 mM glucose) treatment shown by Western blot (a) and its relative densitometric quantification (b). **(F)** The expression of inflammatory markers (IL-6, IL-8 and TNF-α) at the transcriptional level in glucose-treated BPH-1 and WPMY-1 cell lines (0, 25 and 50 mM). **(G)** Immunoblot assay (a) and relative densitometric quantification (b) for IL-6, IL-8 and TNF-α expression in two cell lines undergoing glucose treatment (0, 25 and 50 mM). GAPDH is used as loading control. ns: *p* > 0.05; ^*^ *p* < 0.05; ^**^ *p* < 0.01; ^***^ *p* < 0.001.

In the present work we demonstrated that 25 mM and 50 mM glucose both stimulated prostate cell proliferation, but 50 mM glucose exhibited a more remarkable effect (**Figure 1A**). And this HG-induced stimulation was displayed in a time-dependent manner (**Figure 1A**). We also found that high-concentrations of glucose were able to decrease E-cad and increase N-cad as well as vimentin at transcriptional and translational levels, with 50 mM glucose acting more significantly than 25 mM (*p*<0.05 vs. *p*<0.001) (**Figures 1B, C**). Interestingly, the stimulatory action of 25mM glucose on N-cad protein expression was at a borderline level (*p* = 0.055), although the effect looked very significant (**Figure 1C**(b)). In addition to alterations in expression levels of EMT markers, prostatic cells treated with 25 mM glucose showed higher mRNA and protein expression levels of fibrosis biomarkers (α-smooth muscle actin (α-SMA), collagen-I) (**Figures 1D, E**) and inflammation biomarkers [Interleukin-6 (IL-6), Interleukin-8 (IL-8) and tumor necrosis factor-alpha (TNF-α)] (**Figures 1F, G**) than those cultured in absence of extra glucose (*p*<0.05), while the expression was at much higher level under 50 mM glucose treatment (*p*<0.001) (**Figures 1D–G**).

### 3.3 MID1 might be associated with initiation and progression of BPH among diabetic patients

In view of the reported connection of MID1 molecule with DM and BPH, we determined the expression of MID1 within prostatic cells upon high glucose treatment. Our data illustrated that high-concentration glucose reduced the expression of MID1 level in BPH-1 and WPMY-1 cell lines, also in a dose-dependent manner (**Supplementary Figures S2A, B**).

**Figure 2:**
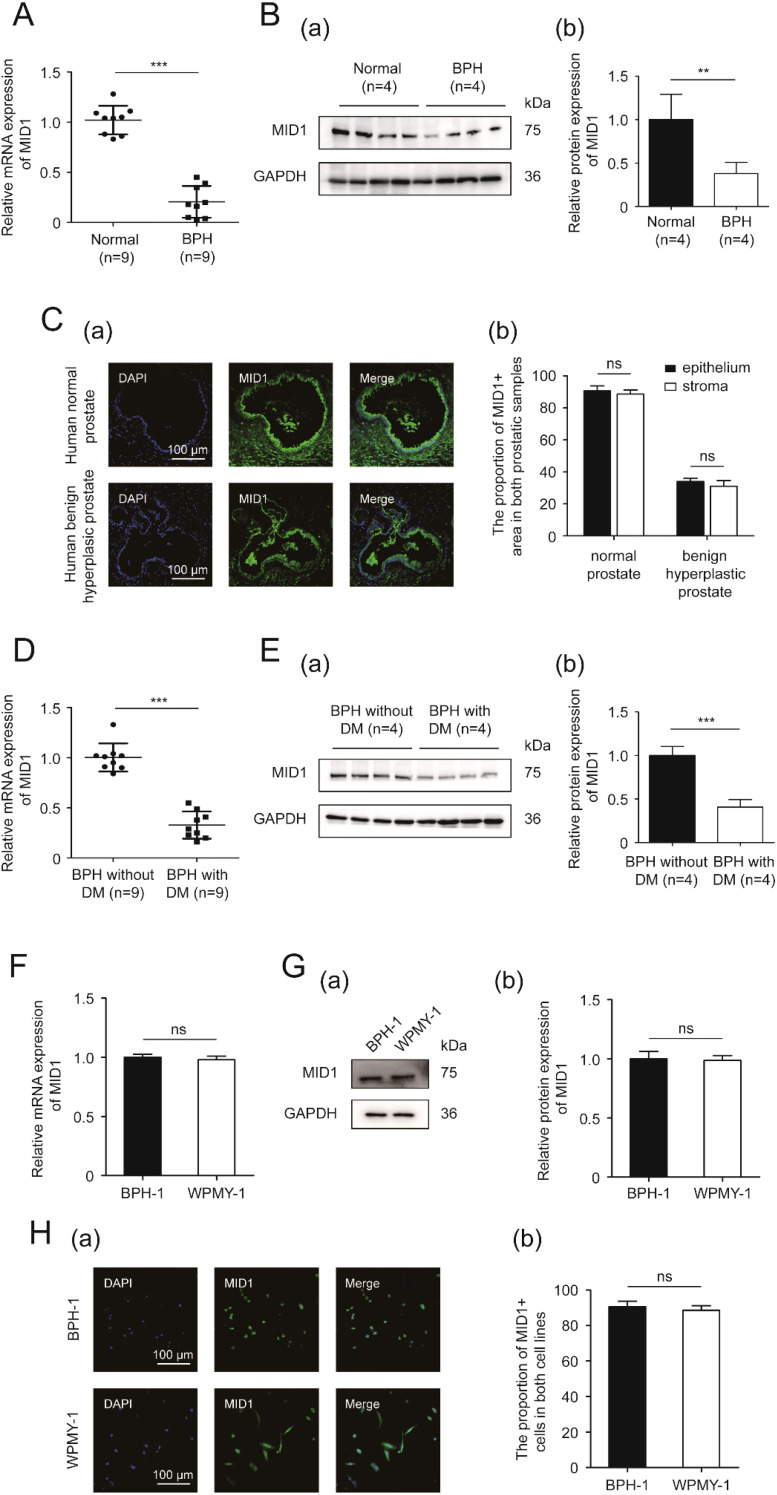
The expression and localization of MID1. **(A)** The expression of MID1 within BPH vs. normal prostatic tissues at the transcriptional level determined by qPCR. **(B)** Western blotting analysis (a) and relative densitometric quantification (b) of MID1 in prostate tissues (BPH vs. normal). **(C)** (a) Immunostaining for MID1 within BPH tissues and normal prostate tissues. DAPI (blue) indicates the nucleus staining. Cy3-immunofluorescence (green) represents MID1 molecule. The scale bars are 100 μm. (b) Quantification for proportion of MID1+ tissues. **(D)** The expression of MID1 within prostatic specimens from diabetic and non-diabetic patients with BPH at the transcriptional level determined by qPCR. **(E)** Western blotting analysis (a) and relative densitometric quantification (b) for MID1 expression levels in BPH tissues (diabetic vs. non-diabetic). **(F)** The mRNA expression of MID1 in BPH-1 cells vs WPMY-1 cells. **(G)** The protein expression and relative densitometric quantification for M1D1 in the two prostate cell lines. **(H)** (a) Immunofluorescence staining for MID1 in BPH-1 and WPMY-1 cell lines. DAPI (blue) indicates the nucleus staining. Cy3-immunofluorescence (green) represents M1D1 staining. The scale bars are 100 μm. (b) The proportion of MID1+ cells was quantified. GAPDH is used as loading control. ns: *p* > 0.05; ^**^ *p* < 0.01; ^***^ *p* < 0.001.

Next, we detected the expression and localization of MID1 within the prostate gland and prostatic cells. Compared with the normal prostate, the hyperplastic tissue showed lower levels of MID1 mRNA and protein expression (**Figures 2A, B**). The immunostaining for MID1 revealed that the fluorescence intensity of the BPH samples was significantly lower than that of normal ones, and this molecule was present in both epithelial and stromal compartments of prostate tissues (**Figure 2C**). Among all prostatic specimens from patients with BPH, those from diabetic individuals employed lower levels of MID1 expression than those from non-diabetic individuals (**Figures 2D, E**). On a cellular level, the two prostate cell lines (BPH-1 and WPMY-1) showed no significant difference in terms of MID1 expression (**Figures 2F, G**). The cellular fluorescence of MID1 within both cell lines was distributed in cytoplasm and on membrane, but not found in nucleus (**Figure 2H**).

### 3.4 MID1 overexpression inhibits cellular proliferation and prevents EMT, fibrosis and inflammation events in prostate cells upon high glucose treatment

**Figure 3:**
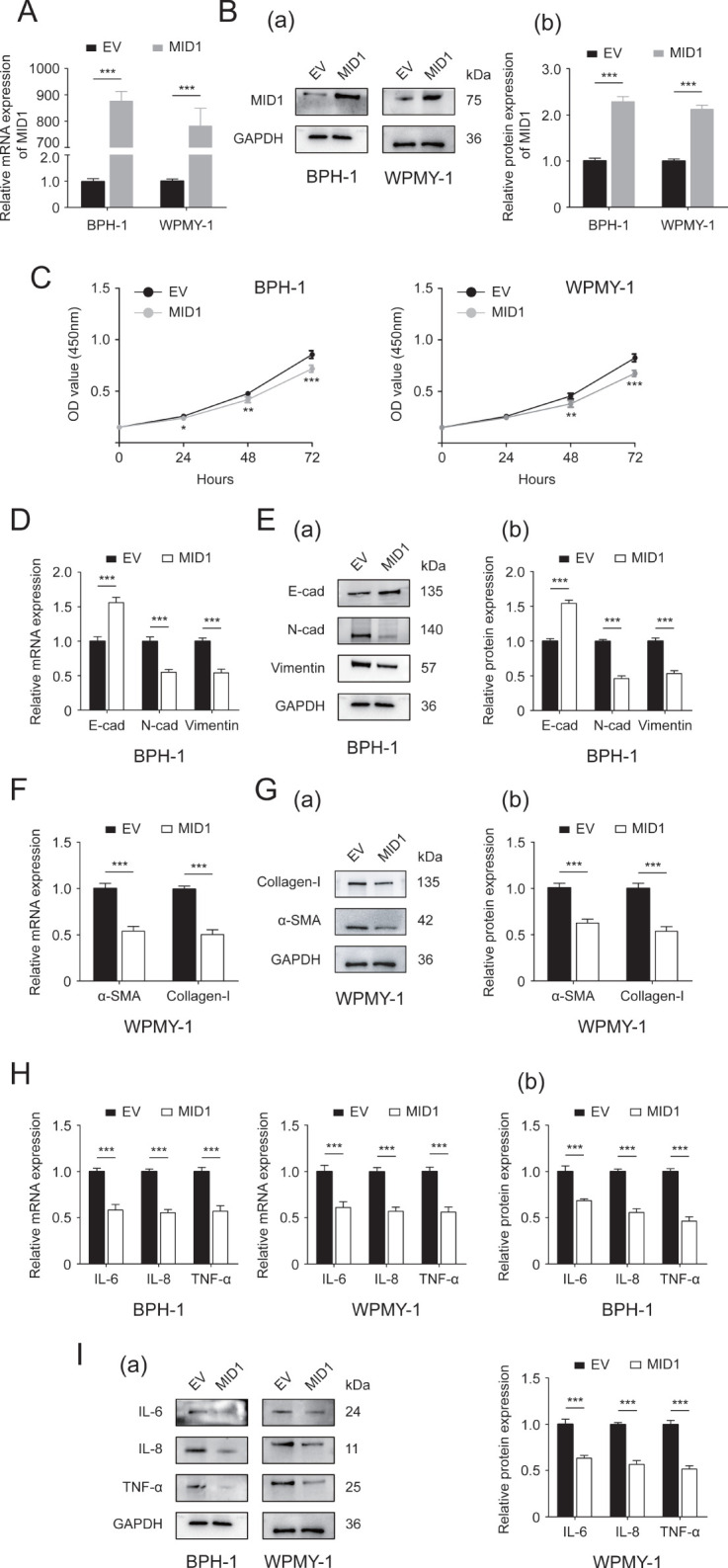
The Effect of MID1 overexpression on the two prostatic cell lines. **(A, B)** The mRNA and protein expression of MID1 in either MID1-overexpressing (MID1) or empty vector (EV)-transposed prostate cell lines (BPH-1 and WPMY-1). **(C)** Cellular proliferative rates at different time points (0, 24, 48and 72h) in BPH-1 and WPMY-1 cells following MID1 plasmid or EV transfection. **(D)** The expression of E-cad, N-cad and vimentin at transcript level in BPH-1 cells with/without MID1 overexpression. **(E)** The protein expression of EMT biomarkers in BPH-1 cell line upon MID1 overexpression (+)/(-) shown by Western blot (a) and its relative densitometric quantification (b). **(F)** The mRNA expression ofα-SMA and collagen-I in WPMY-1 cells following MID1 overexpression (+)/(-). **(G)** Immunoblot assay (a) and relative densitometric quantification (b) for two fibrosis markers in MID1 plasmid-/EV-transposed WPMY-1 cells. **(H, I)** The expression of inflammatory markers IL-6, IL-8 and TNF-α at transcriptional and translational levels in BPH-1 and WPMY-1 cells with/without MID1 overexpression. GAPDH is used as loading control. ^*^ *p* < 0.05; ^**^ *p* < 0.01; ^***^ *p* < 0.001.

In an attempt to investigate the function of MID1, we established prostate cell lines overexpressing MID1 using pcDNA3.1- MID1. We initially compared the expression of MID1 levels between MID1-overexpressing cells and empty vector-transposed cells. Our results demonstrated that MID1 overexpression significantly promoted the expression of MID1 (both at mRNA and protein levels) within BPH-1 and WPMY-1 cell lines (**Figures 3A, B**). Further, the CCK8 data illustrated that overexpression of MID1 dramatically decreased proliferative rates of two prostatic cell lines (**Figure 3C**). MID1 overexpression also downregulated N-cad, vimentin, αSMA, collagen-I, IL-6, IL-8 and TNF-α, in BPH-1 and WPMY-1 cells, as confirmed by qPCR and Western blot analysis (**Figures 3D–I**). In contrast, E-cad was upregulated at both mRNA and protein levels within two cell lines upon MID1 overexpression (**Figures 3D, E**).

**Figure 4:**
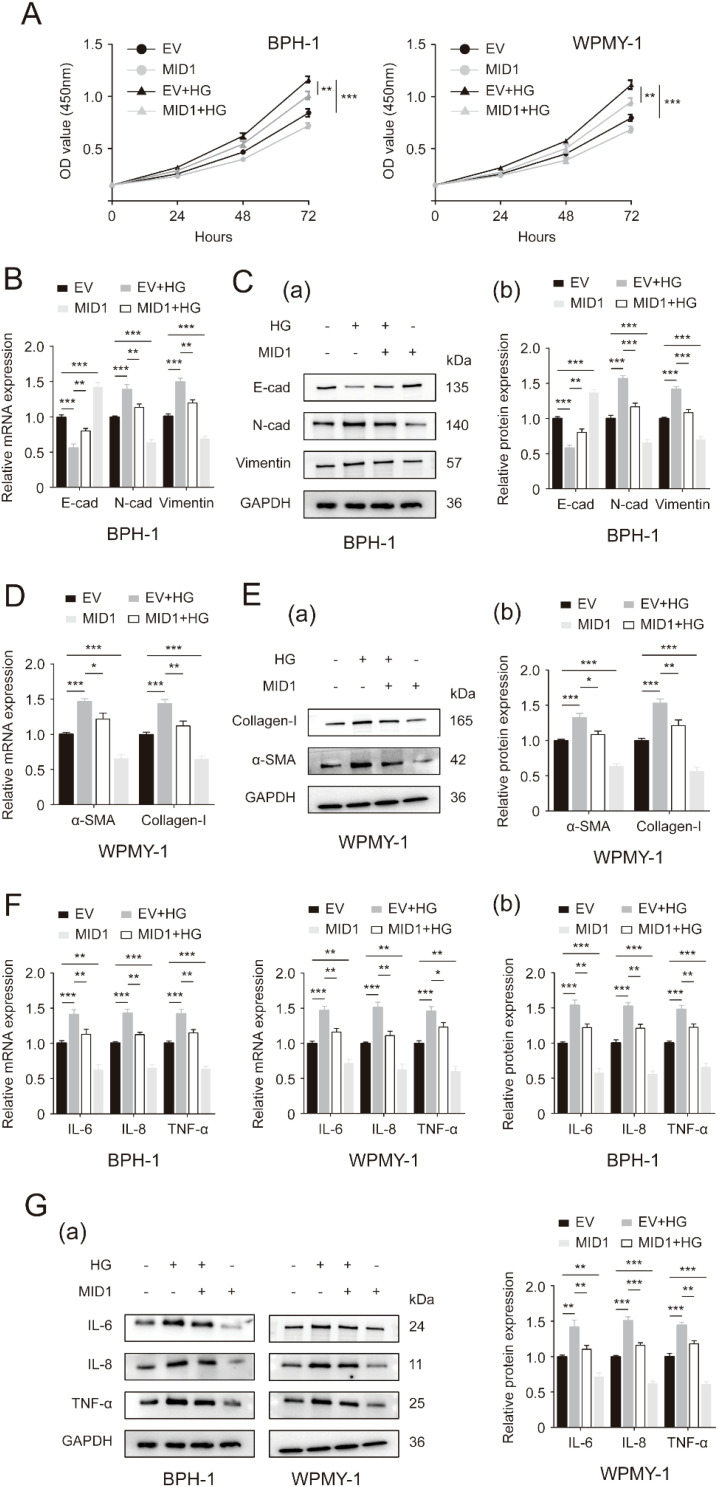
MID1 overexpression abrogates high glucose-induced changes in proliferative rates, as well as EMT, fibrosis and inflammation status of prostatic cells. **(A)** Cellular proliferative rates of BPH-1 and WPMY-1 cell lines at different time points (0, 24, 48and 72h) upon MID1 overexpression (+)/(-) and high glucose (HG) treatment (+)/(-). **(B)** The mRNA expression of E-cad, N-cad and vimentin in BPH-1 cell line undergoing MID1 plasmid/empty vector (EV) transfection and HG treatment (+)/(-). **(C)** The expression of three EMT biomarkers at the translational level in BPH-1 cells under the same experimental conditions shown by Western blot (a) and its relative densitometric quantification (b). **(D)** The expression ofα-SMA and collagen-I at the transcriptional level in WPMY-1 cells following MID1 overexpression (+)/(-) and HG treatment (+)/(-). **(E)** Immunoblot assay (a) and relative densitometric quantification (b) for two fibrosis molecules in WPMY-1 cells under the same experimental conditions. **(F, G)** The mRNA and protein expression of inflammatory markers in BPH-1 and WPMY-1 cells subjected to MID1/EV transfection and HG treatment (+)/(-). GAPDH is used as loading control. ^*^ *p* < 0.05; ^**^ *p* < 0.01; ^***^ *p* < 0.001.

Next, we used either pcDNA3.1-MID1 or empty vector to treat BPH-1 and WPMY-1 cell lines, followed by 50 mM glucose treatment or remaining left untreated. As expected, the expression of MID1 levels decreased in HG-treated prostatic cells, and MID1 overexpression significantly increased its expression levels (**Supplementary Figures S2C, D**). Our data further showed that high-concentration glucose stimulated cellular proliferation of BPH-1 and WPMY-1 cells, and this stimulation could be revoked by pcDNA3.1-MID1 transfection (**Figure 4A**). Additionally, the MID1 plasmid transfection was shown to recover HG-induced downregulation of E-cad and upregulation of N-cad as well as vimentin in two cell lines (**Figures 4B, C**). In HG-treated cells the increase in the expression of fibrosis markers αSMA and collagen-I, as well as inflammation markers IL-6, IL-8 and TNF-α, was also corrected by MID1 overexpression (**Figures 4D–G**).

### 3.5 PP2A as a potential downstream target of MID1

**Figure 5:**
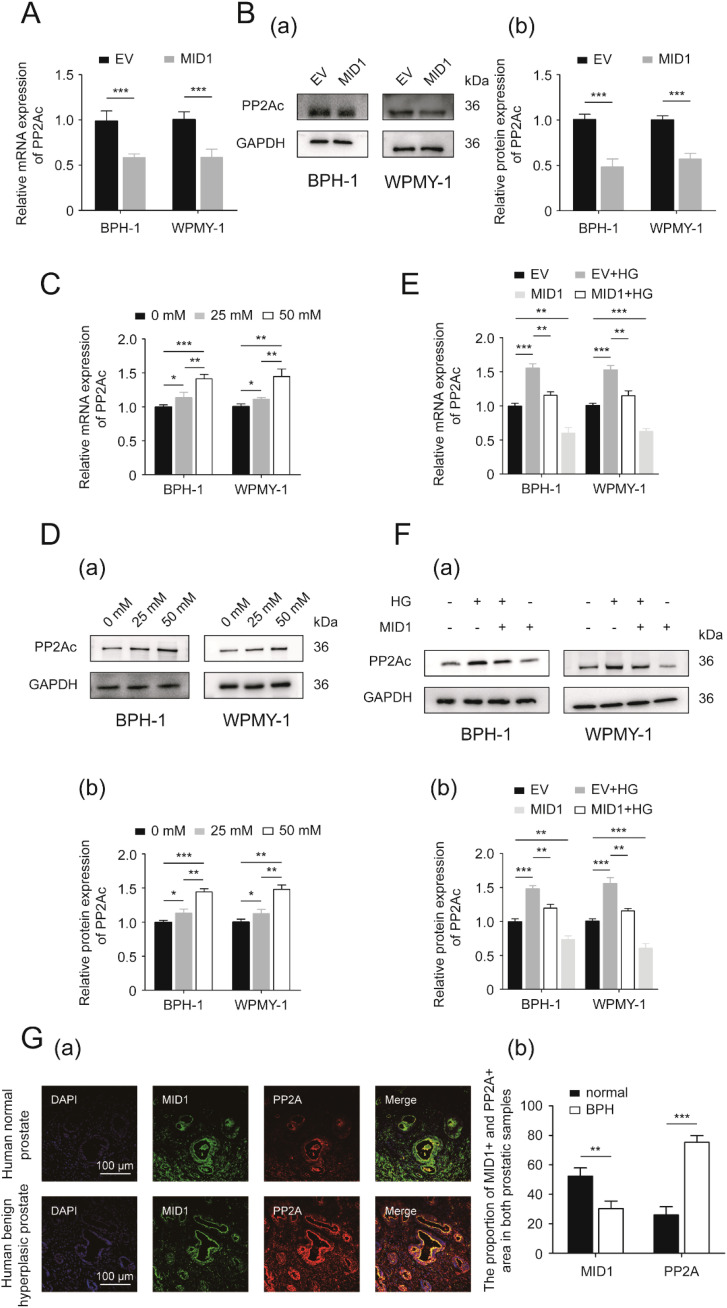
PP2A might be the downstream target of MID1. **(A)** The expression of PP2Ac (protein phosphatase 2A catalytic subunit) at the transcriptional level in BPH-1 and WPMY-1 cell lines following empty vector/MID1 plasmid transfection. **(B)** Western blot analysis (a) and relative densitometric quantification (b) for PP2Ac in either MID1-overexpressing or empty vector (EV)-transposed prostatic cell lines (BPH-1 and WPMY-1). **(C, D)** The mRNA and protein expression of PP2Ac in high glucose (HG)-treated prostatic cells. **(E, F)** The expression of PP2Ac at transcriptional and protein levels in the two prostatic cell lines upon MID1 overexpression (+)/(-) and HG treatment (+)/(-). **(G)** (a) Immunofluorescence staining for MID1 and PP2Ac in normal and hyperplastic prostate. DAPI (blue) indicates nucleus staining. Cy3-immunofluorescence (green and red) represents MID1 and PP2Ac protein staining, respectively. The scale bars are 100 μm. (b) Quantification for proportion of MID1+ and PP2Ac + prostatic tissues. GAPDH is used as loading control. ^*^ *p* < 0.05; ^**^ *p* < 0.01; ^***^ *p* < 0.001.

Since PP2A is one of the most important molecular targets of MID1 and plays a critical role in the process of MID1 exerting its function, we determined the expression of PP2Ac, the critical functional subunit of PP2A molecule, within either MID1-overexpressing or empty vector-transposed cells. We observed that in both cell lines (BPH-1 and WPMY-1) MID1 overexpression decreased PP2Ac (**Figures 5A, B**), high-concentration glucose enhanced the expression of PP2Ac level in a dose-dependent manner (**Figures 5C, D**), and HG-induced reduction of PP2Ac was abrogated by MID1 overexpression (**Figures 5E, F**). Additionally, immunofluorescence images illustrated PP2Ac was at higher level in hyperplasic prostates than in normal ones and was co-localized with MID1 within the prostate gland (**Figure 5G**).

### 3.6 WNT/β-catenin signaling might be the underlying mechanism by which loss of MID1 led to EMT, fibrosis and inflammation in prostate cells undergoing high glucose treatment

We further investigated through which downstream signaling MID1 inhibited EMT, inflammation and fibrosis in HG-treated prostate cell lines. Considering the important role of WNT/β-catenin signaling in regulating EMT, inflammation and fibrosis in many cell lines, we first determined the expression of β-catenin levels in prostate cell lines (BPH-1 and WPMY-1) in response to HG treatment. It was revealed in the present work that 25 mM glucose increased the mRNA and protein expression levels of β-catenin in two prostatic cell lines, while 50 mM glucose could further promote its expression (**Supplementary Figures S3A, B**). Our data also showed that MID1 overexpression downregulated β-catenin (**Supplementary Figures S3C, D**) and abrogated HG-induced decrease of this molecule(**Supplementary Figures S3E, F**). Meanwhile, the phosphorylation level of β-catenin was elevated in two cell lines following MID1 overexpression (**Supplementary Figures S3C, D**).

**Figure 6:**
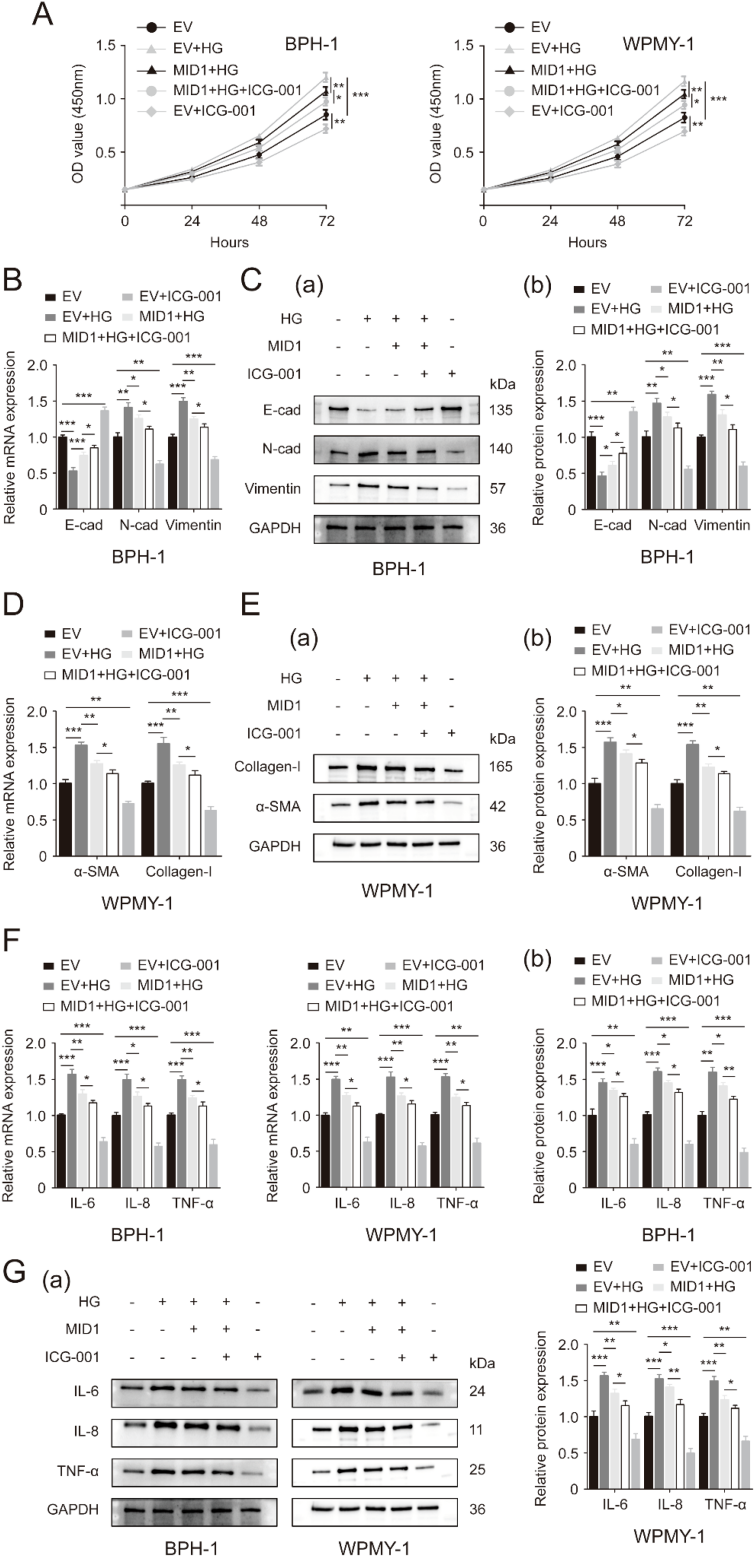
Inactivation of WNT/β-catenin cascade exacerbates the inhibitory effects of MID1 overexpression on high glucose-induced alterations within prostatic cells. **(A)** Proliferative rates of BPH-1 and WPMY-1 cell lines at different time points (0, 24, 48, 72h) upon MID1 overexpression (+)/(-), high glucose (HG) treatment (+)/(-) and the β-catenin inhibitor ICG-001 (+)/(-). **(B, C)** The mRNA and protein expression of E-cad, N-cad and vimentin in the BPH-1 cell line undergoing above-mentioned experimental treatment. **(D, E)** The expression of α-SMA and collagen-I at the transcriptional and translational levels in WPMY-1 prostatic cells following MID1 overexpression (+)/(-), as well as HG (+)/(-) and ICG-001 (+)/(-) treatment. **(F, G)** The mRNA and protein expression of inflammatory markers IL-6, IL-8 and TNF-α in BPH-1 and WPMY-1 cells. EV, empty vector. GAPDH is used as loading control. ^*^ *p* < 0.05; ^**^ *p* < 0.01; ^***^ *p* < 0.001.

Then, we used a β-catenin inhibitor named ICG-001 to inactivate WNT/β-catenin signaling. Detection of cell proliferation of two prostatic lines by CCK8 assay demonstrated that addition of 4 μM ICG-001 drastically decreased the cellular proliferative rates of both lines while 5 μM of ICG-001 was not more significantly effective compared to 4 μM (**Supplementary Figure S4**). In the following experiments, therefore, we treated two prostate cell lines subjected to high glucose pretreatment and MID1 transfection with 4 μM ICG-001. ICG-001 at this experimental dose was confirmed to further decrease β-catenin in the two prostatic lines undergoing HG treatment and MID1 overexpression (**Supplementary Figures S3G, H**). As expected, ICG-001 has no impact on MID1 and PP2Ac expression levels (**Supplementary Figures S2E, F**). Our present CCK8 data revealed that high glucose treatment stimulated cellular proliferation of BPH-1 and WPMY-1 cell lines and MID1 overexpression could inhibit this stimulatory effect, while compared with cells under high glucose pretreatment and MID1 overexpression additional ICG-001 treatment could further decrease the proliferative rate of two cell lines (**Figure 6A**). In addition to cell proliferation, ICG-001 could also impair the effects of MID1 overexpression in terms of inflammation, fibrosis and EMT on the two lines: high glucose-induced either up- or downregulation of those molecular markers was recovered in cells overexpressing MID1, and this recovery could be further intensified by ICG-001 (**Figures 6B–G**).

### 3.7 Prostate volume and IPSS statistically correlate with expression of MID1 and PP2Ac

**Figure 7:**
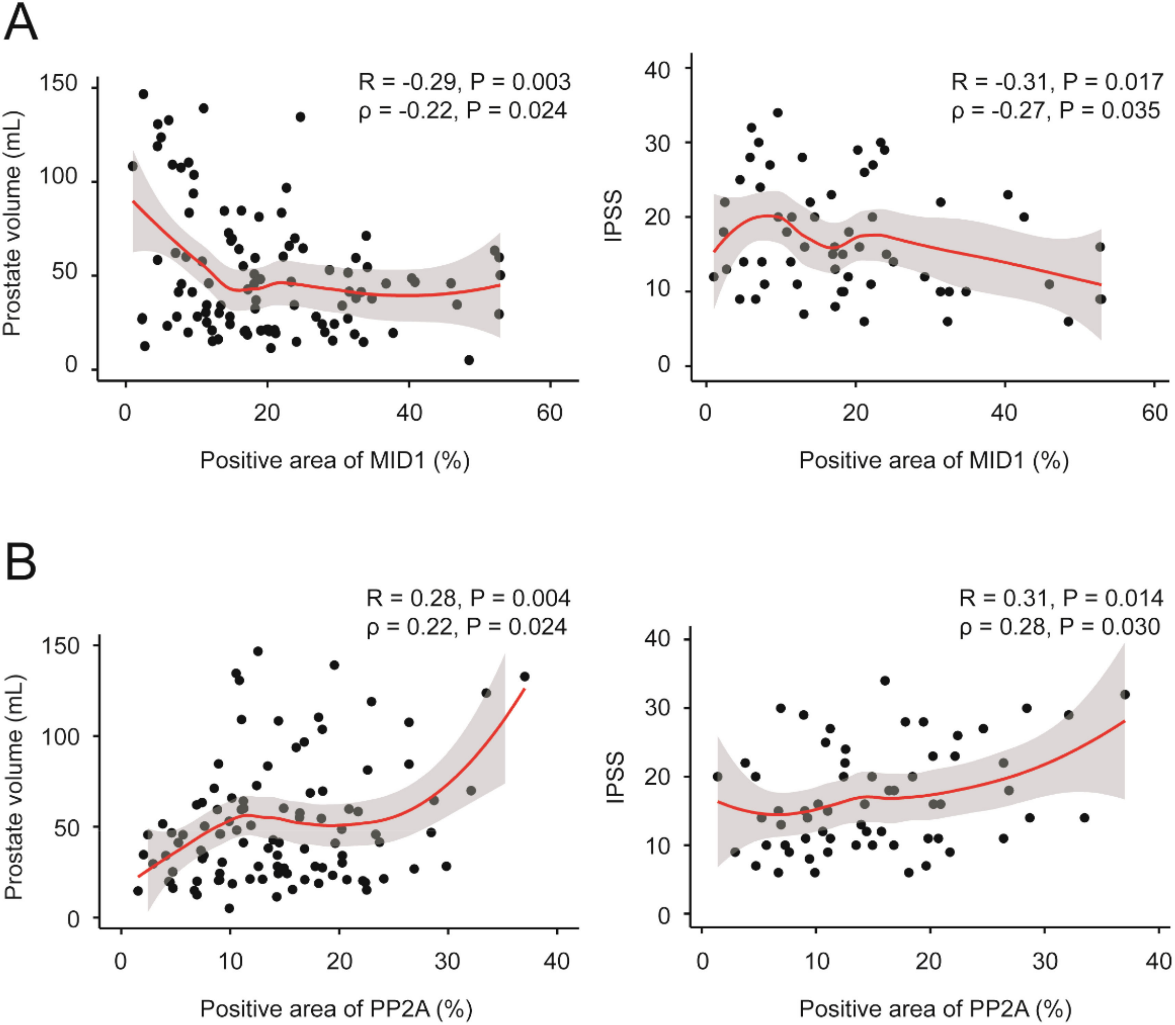
Correlation between MID1, PP2Ac and multiple clinical parameters. **(A)** Prostate volume and IPSS negatively correlated with intraprostatic MID1 levels. Loess regression curves were overlaid on the scatter plots. **(B)** Prostate volume and IPSS positively correlated with intraprostatic PP2Ac levels. Loess regression curves were overlaid on the scatter plots.

Finally, we investigated correlation between MID1, PP2Ac and multiple clinical parameters by Pearson and Spearman’s rank correlation analysis. Although significant correlation was observed in terms of two genes of interest MID1 & PP2Ac and two clinical parameters PV & IPSS in Pearson correlation analysis (**Table 2**), evident heteroscedasticity reported in **Figure 7A** indicated that Spearman’s rank correlation analysis seemed to be a better choice. Our data demonstrated that prostate volume significantly correlated with prostatic levels of MID1 and PP2Ac (negatively and positively, respectively) (**Tables 2**, **3**, **Figure 7A**). IPSS scores also showed negative correlation with intraprostatic MID1 and positive correlation with PP2Ac expression (**Tables 2**, **3**, **Figure 7B**). No statistically significant correlation was observed between other clinical parameters (e.g. BMI, Qmax) and intraprostatic levels of MID1 and PP2Ac expression (**Table 2**).

**Table 2:** Analysis for Pearson correlation between genes of interest and clinical parameters.

Clinical parameters	MID1		PP2Ac	
	Pearson Correlation	*p* value	Pearson Correlation	*p* value
BMI (kg/m^2^)	-.09	.389	.02	.860
FBG (mmol/L)	-.13	.195	.11	.152
HbA1c (%)	-.16	.094	.15	.088
PV (mL)	-.29	.003**	.28	.004**
fPSA (ng/mL)	-.09	.363	.09	.387
tPSA (ng/mL)	-.14	.146	.07	.496
f/t	.05	.589	.03	.761
IPSS	-.31	.017*	.31	.014*
Qmax (mL/s)	.19	.063	-.05	.704

BMI, body mass index; FBG, fasting blood glucose; PV, prostate volume; tPSA, total prostate specific antigen; fPSA, free prostate specific antigen; t/f, tPSA/fPSA; IPSS, international prostate symptom score; Qmax, maximum urine flow rate. ^*^ *p* < 0.05; ^**^ *p* < 0.01.

**Table 3:** Analysis for Spearman correlation between genes of interest and clinical parameters.

Clinical parameters	MID1		PP2Ac	
	Spearman Correlation	*p* value	Spearman Correlation	*p* value
PV (mL)	-.22	.024*	.22	.024*
IPSS	-.27	.035*	.28	.030*

PV, prostate volume; IPSS, international prostate symptom score. ^*^ *p* < 0.05.

## 4 Discussion

Our present work revealed that HG treatment inhibited the expression of MID1, thus stimulating the proliferation of prostatic cells and triggering EMT, fibrosis and inflammation within two prostatic cell lines via increase of PP2Ac and resultant enhanced WNT/β-catenin signaling. From the perspective of clinical parameters, diabetic individuals with BPH had larger prostate volume, higher IPSS scores and lower Qmax than non-diabetic groups, which was probably attributed to HG-induced EMT, fibrosis and inflammation events. Additionally, intraprostatic MID1 and PP2Ac levels statistically correlated with prostate volume and IPSS scores.

The relationship between DM and BPH has been documented in published literature. Numerous clinical studies have shown that individuals with higher fasting blood glucose levels tend to have larger prostate glands, higher IPSS and an increased risk of BPH; on the other hand, lowering the blood glucose level can attenuate the progression of BPH (7, 8, 19). This is compatible with our observations that the prostate volume was larger and IPSS was higher in men with BPH complicated by DM. Here comes a question: why does high glucose concentration increase the prostate volume and the risk of BPH? One of the explanations for this is that HG-induced prostate inflammation results in the increase of prostate volume – an important parameter associated with urethral resistance. Published data from the literature has demonstrated oxidative stress resulting from metabolic disorders of glucose can activate nuclear factor-kappa B (NF-κB), elevating the expression of pro-inflammatory cytokines, such as TNF-α, interleukin-1 beta (IL-1β) and IL-6 (12). The production and action of these pro-inflammatory cytokines further cause the generation of ROS, thereby potentiating this positive feedback loop (20). Meanwhile, the correlation between the degree of prostatic inflammation and prostate size is significant (21). The molecular mechanism underlying this correlation is that chronic inflammatory conditions favor the release of cytokines; this action promotes the production of growth factors that are able to stimulate the proliferation of prostate cells (22). In line with this concept, our novel results illustrate that prostate cell lines exposed to high glucose tended to proliferate more rapidly and had higher expression levels of inflammatory cytokines (IL-6, IL-8 and TNF-α). Actually, a newly-published article by Wei and his colleagues also reported an increase in proliferative rates of BPH-1 and RWPE-1 cell lines upon high glucose treatment (9), strongly supporting the intimate relationship between HG and enhanced proliferation of prostatic cells. Moreover, HG-induced prostate inflammation and fibrosis are documented to cause increased prostate rigidity and ultimately bladder outlet obstruction (13). In the present study we found that the Qmax was significantly lower in diabetic patients with BPH than non-diabetics. We further revealed that HG induced fibrosis in two prostate cell lines, as shown by elevated expression levels of fibrosis biomarkers following 25 and 50 mM glucose treatment. Such results first establish a connection between high-concentration glucose and fibrosis within the prostate gland. Growing evidence has suggested a significant role of EMT in the development of BPH, providing a new understanding into the origin of prostatic stroma (11, 23–26). It was interesting to observe that high levels of glucose could activate the EMT event within prostatic cell lines (9, 10). This indicates that HG-induced EMT is also a cause of the initiation and progression of BPH among diabetic patients. Our data provided evidence for this view, demonstrating that high glucose treatment on two prostate cell lines decreased E-cad and increased N-cad as well as vimentin at both mRNA and protein levels.

There have been numerous genetic alterations detected in diabetic patients, among which a gene named MID1 is likely to be associated with high HG-induced BPH. MID1, also called TRIM18, is one of the members of TRIM family and has E3 ubiquitin ligase activity (15). By inactivating the downstream molecule PP2A, MID1 could hinder the progression of prostate inflammation, fibrosis and EMT (16–18). Interestingly, high levels of glucose stimulated MID1 expression in renal cell lines (HK-2) and elevated MID1 expression favored the activation of EMT, inflammation as well as fibrosis (18), suggesting the association between MID1 expression levels and glucose concentration on the one hand, and three important pathophysiological processes: EMT, inflammation and fibrosis on the other. However, this conclusion is inconsistent with our present findings showing that hyperplastic prostate tissues possessed lower levels of MID1 expression than normal ones. This difference may derive from the different functions that MID1 have in different organs. An important piece of supporting evidence for our speculation is the cancer data from GEPIA (http://gepia.cancer-pku.cn/) demonstrating that the MID1 gene had a different expression profile in prostatic and renal tissue [the expression of MID1 in renal carcinoma vs. in normal tissue is 10.9 vs. 7.5 (kidney renal clear cell carcinoma, KIRC) and 6.53 vs. 5.01 (kidney renal papillary cell carcinoma, KIRP), but in prostate carcinoma vs. in normal tissue is 4.51 vs. 7.95 (PRAD)]. Thus, it is probably true that MID1 is an anti-oncogene gene in the prostate, different from its function as a carcinomic gene in renal tissue. In our study, we did find MID1 overexpression led to downregulation of EMT, inflammation and fibrosis biomarkers except for E-cad in two prostate cell lines. And overexpression of MID1 could impede the progression of HG-induced EMT, inflammation and fibrosis, as demonstrated by the observation that upregulation of MID1 impaired the impacts of high glucose treatment on those molecular markers. On the other hand, among patients with BPH, those with concomitant DM had lower MID1 levels than those without, and HG treatment decreased MID1 expression levels in prostatic cells, which strongly supports its intimate relationship with diabetes. Because of the importance of PP2A in the process by which MID1 exerts its function, we further detected the expression of PP2Ac – the catalytic subunit of PP2A, the potential downstream target of MID1 – in prostatic cells following either HG treatment or MID1 overexpression. Our novel data illustrated that MID1 overexpression inhibited PP2Ac expression, HG treatment elevated the expression of PP2Ac, and this stimulatory effect of high glucose could be abrogated by MID1 overexpression. Moreover, immunofluorescence images illustrated PP2Ac was at a higher level in hyperplasic prostates than in normal ones and was co-localized with MID1 within the prostate gland. All these results suggest MID1 could inhibit high glucose-induced EMT, fibrosis and inflammation within the prostate gland probably by inhibiting PP2A expression.

We further investigated the molecular mechanism behind the inhibitory effect of MID1. Until now, a number of molecular pathways have been pointed out to take part in the origin and progression of EMT, inflammation and fibrosis, such as canonical & noncanonical WNT/β-catenin cascade (27, 28), NF-κB signaling (29, 30) and PI3K/AKT/mTOR molecular pathway (31–33), among which WNT/β-catenin signaling cascade is the most frequently cited and important one. For example, available literature has reported the implication of WNT/β-catenin signaling in those three events within prostate gland (27, 34). It is worthy of note that PP2A has been shown to regulate WNT/β-catenin signaling, either by dephosphorylating β-catenin and therefore enhancing the signal or by binding to the destruction complex through APC or associating with GSK3β to inhibit this pathway (35). Given the association of this signaling cascade with EMT, inflammation and fibrosis, as well as its molecular connection with MID1/PP2A, we determined the expression levels of its molecular markers in prostate cells undergoing high glucose treatment and MID1 overexpression. Our data demonstrated that in two prostatic cell lines HG treatment stimulated the expression of β-catenin levels while MID1 overexpression downregulated β-catenin. Furthermore, MID1 overexpression promoted phosphorylation of β-catenin. This result indicates that MID1 regulates WNT/β-catenin signaling by impacting phosphorylation status of β-catenin. Then, we treated cells with the β-catenin inhibitor ICG-001, after determining the experimental dose, and observed the outcomes of inactivating the WNT/β-catenin molecular pathway. As expected, ICG-001 was able to inactivate this signaling, as demonstrated by downregulation of β-catenin, and to further potentiate the impact of MID1 overexpression on all biomarkers (E-cad, N-cad, vimentin, α-SMA, collagen-I, IL-6, IL-8 and TNF-α). This indicates that WNT/β-catenin cascade is a critical molecular pathway, the inhibition of which was associated with the inhibitory effect of MID1 on HG-induced EMT, inflammation and fibrosis in prostate cell lines.

We finally investigated the correlation between MID1, PP2Ac and multiple clinical parameters by Pearson correlation and Spearman’s rank analysis. We found that prostate volume negatively and positively correlated with intraprostatic MID1 and PP2Ac expression, respectively. This was also the case with IPSS. No statistically significant correlation was observed between other clinical parameters (e.g. BMI, Qmax) and prostatic levels of MID1 and PP2Ac genes.

However, we have to admit that there exist some limitations in this work. Diabetes is a complex metabolic disorder, and many contributing factors, such as hyperinsulinemia and insulin resistance, have been involved in the development of BPH under diabetic conditions (36). Whether such pathophysiological changes are related to changed MID1 expression profile was not intensively studied in this work and is still unclear. Moreover, our study relies on *in vitro* cell models and *in vivo* validation is not available in this work. In order to strengthen our findings, using animal models to validate our experimental results is necessary. This might be one of the future research directions concerning the association between MID1 and BPH.

## 5 Conclusion

**Figure 8:**
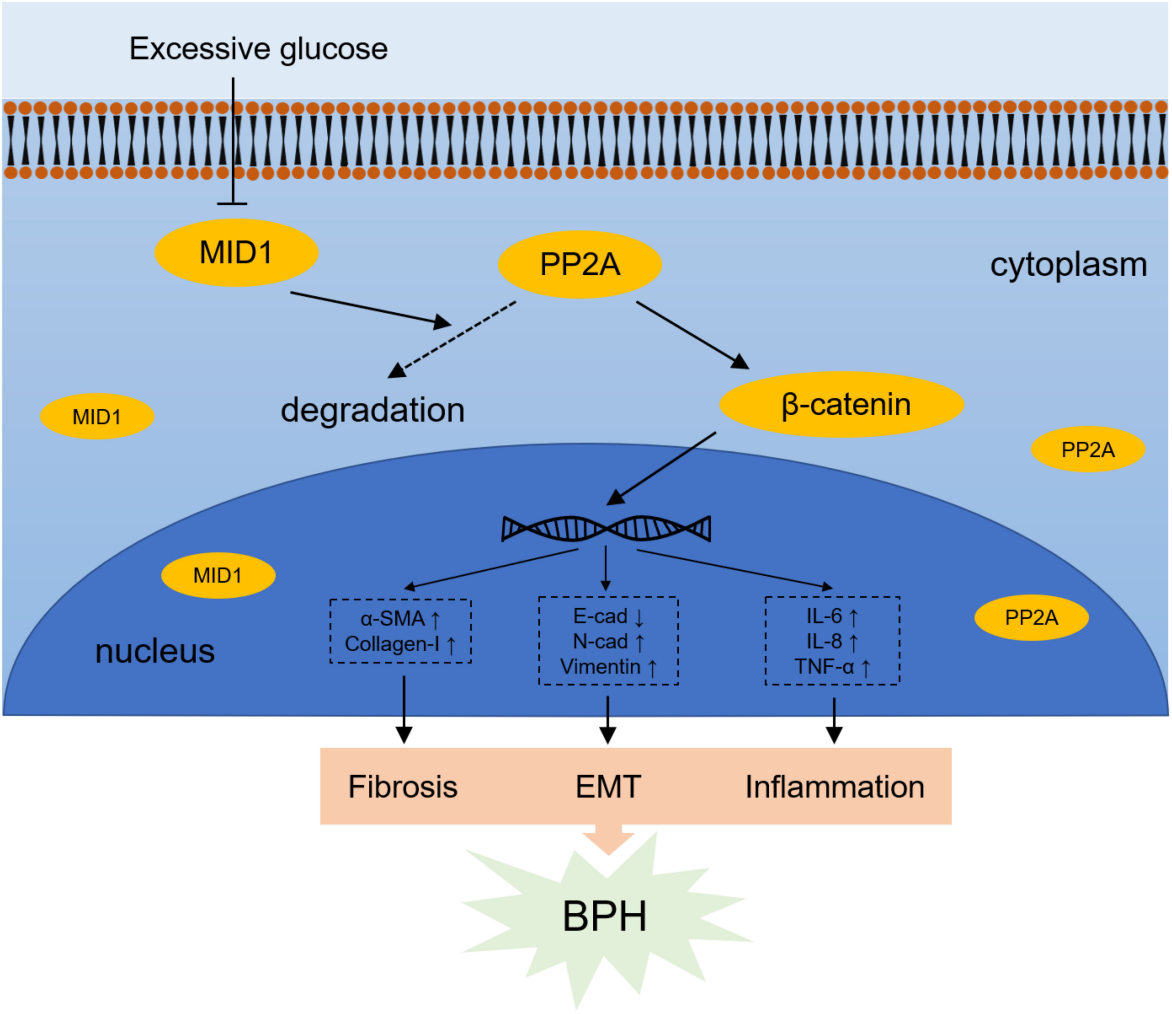
Schematic illustration for mechanism by which high glucose-induced MID1 downregulation results in the origin of fibrosis, EMT and inflammation within prostatic cells. High concentration of glucose reduces the expression of MID1, reducing the degradation of PP2A and therefore leading to its accumulation in the cytoplasm. Elevated levels of PP2Ac (the catalytic subunit of PP2A) results in upregulation and dephosphorylation of β-catenin and resultant increase of downstream WNT responsive genes.

In aggregate, we uncovered the effect of high glucose treatment on prostatic cells. Prostatic inflammation, fibrosis and EMT are the molecular basis bridging BPH and diabetes: HG treatment induced downregulation of MID1 and upregulation of PP2A, thus promoting progression of the three biological events within prostatic cell lines via enhanced WNT/β-catenin signaling (**Figure 8**). Targeting MID1 appears to be an emerging area to impede prostate enlargement, rigidity increase and LUTS progression.

## Data Availability

The raw data supporting the conclusions of this article will be made available by the authors, without undue reservation.

## Supplementary material

Supplementary Figure 1 Determination of experimental dose of glucose for treatment of prostatic cell lines. Cell viability of BPH-1 and WPMY-1 cell lines upon treatment with increasing concentrations of glucose (0, 12.5, 25, 37.5, 50 and 62.5 mM) for 48 h. ns: *p* > 0.05; **p* < 0.05; ****p* < 0.001.

Supplementary Figure 2 The expression of MID1 and PP2A levels in prostatic cells upon various experimental treatments. **(A)** The mRNA expression of MID1 in BPH-1 and WPMY-1 cells undergoing 0, 25 and 50 mM glucose treatment. **(B)** Immunoblot assay (a) and relative densitometric quantification (b) for MID1 expression in high glucose (HG)-treated two prostatic cell lines. **(C, D)** The expression of MID1 at transcriptional and translational levels in BPH-1 and WPMY-1 cells upon HG treatment (+)/(/) and MID1 plasmid/EV transfection shown by qPCR **(C)**, as well as western blot **(D** (a)) and relative densitometric quantification **(D** (b)). **(E, F)** The mRNA and protein expression of MID1 and PP2A levels in prostatic cell lines treated with MID1 overexpression (+)/(-), high glucose (+)/(-) and ICG-001 (+)/(-). GAPDH is used as loading control. ns: *p* > 0.05; **p* < 0.05; ***p* < 0.01; ****p* < 0.001.

Supplementary Figure 3 The status of WNT/β-catenin signaling within two prostate cell lines upon various experimental treatments. **(A)** The mRNA and protein expression of β-catenin levels in prostatic cell lines (BPH-1 and WPMY-1) treated with increasing glucose concentrations (0, 25, 50 mM). **(B)** Immunoblot assay (a) and relative densitometric quantification (b) for expression of β-catenin in two prostatic cell lines following 0, 25, 50 mM glucose treatment. **(C, D)** The expression of β-catenin and phospho-β-catenin (ser33/37/thr41 & thr41/ser45) at transcriptional and translational levels in either MID1 plasmid- or empty vector (EV)- transposed prostate cell lines. **(E, F)** The mRNA and protein expression of β-catenin in BPH-1 and WPMY-1 cell lines upon MID1 overexpression (+)/(-) and high glucose treatment (+)/(-). **(G, H)** The expression of β-catenin at both mRNA and protein levels in two prostatic cell lines treated with MID1 overexpression (+)/(-), high glucose (+)/(-) and ICG-001 (+)/(-). GAPDH is used as loading control. **p* < 0.05; ***p* < 0.01; ****p* < 0.001.

Supplementary Figure 4 Determination of experimental dose of ICG-001 for treatment of prostatic cell lines. Cell viability of BPH-1 and WPMY-1 cell lines upon treatment with increasing concentrations of ICG-001 for 48 h. ns: *p* > 0.05; **p* < 0.05; ***p* < 0.01; ****p* < 0.001.
