# Supplementary figures and images for: Midline-1 inhibited high glucose-induced epithelial-mesenchymal transition, fibrosis and inflammation through WNT/β-catenin signaling in benign prostatic hyperplasia
Source: Front Endocrinol (Lausanne). 2025 Mar 26;16:1543295. doi: 10.3389/fendo.2025.1543295 (PMC11978649; doi:10.3389/fendo.2025.1543295)

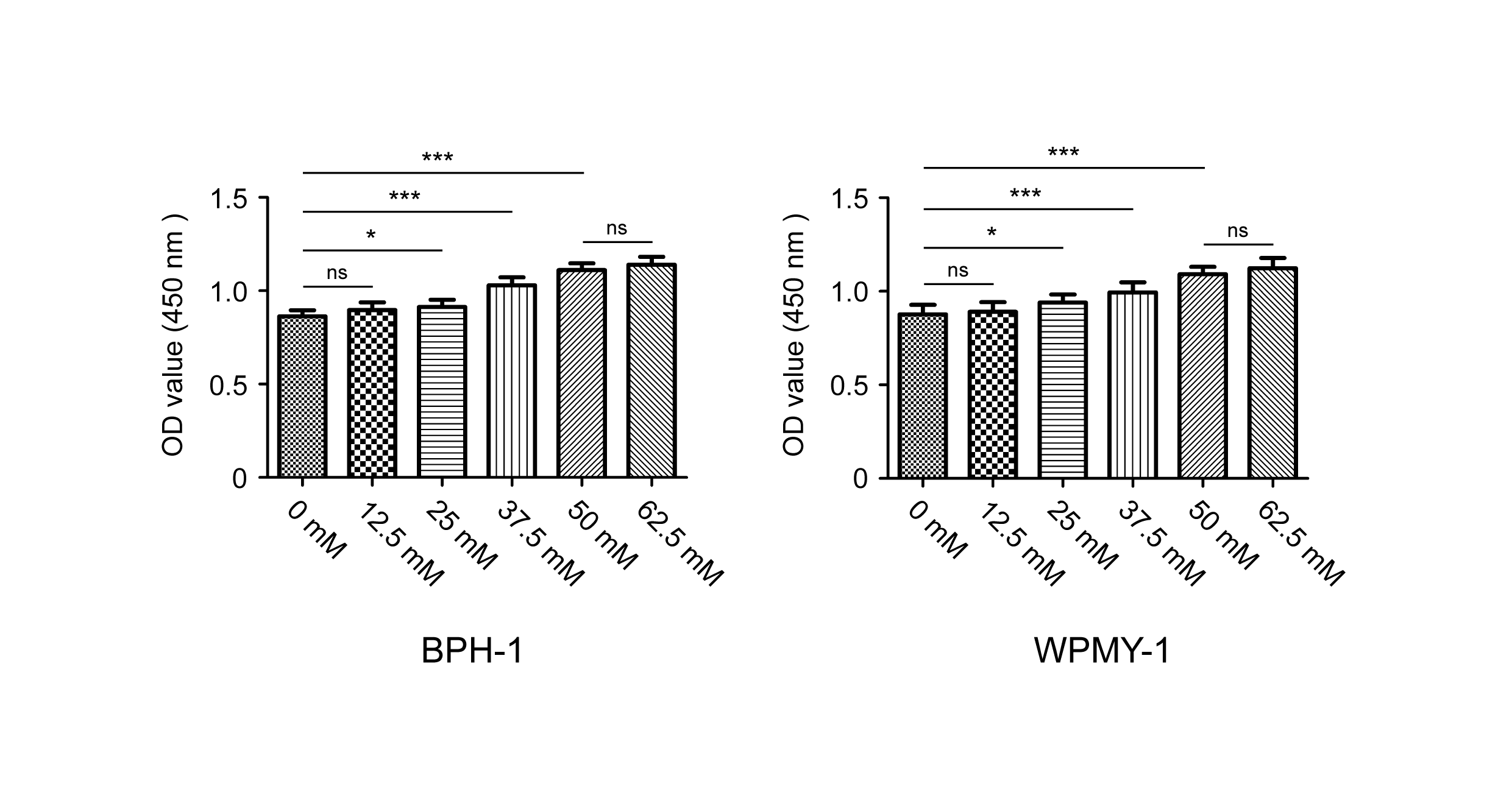

Supplement: Supplementary Figure 1 — Determination of experimental dose of glucose for treatment of prostatic cell lines. Cell viability of BPH-1 and WPMY-1 cell lines upon treatment with increasing concentrations of glucose (0, 12.5, 25, 37.5, 50 and 62.5 mM) for 48 h. ns: p > 0.05; *p < 0.05; ***p < 0.001. [file Image1.tif]

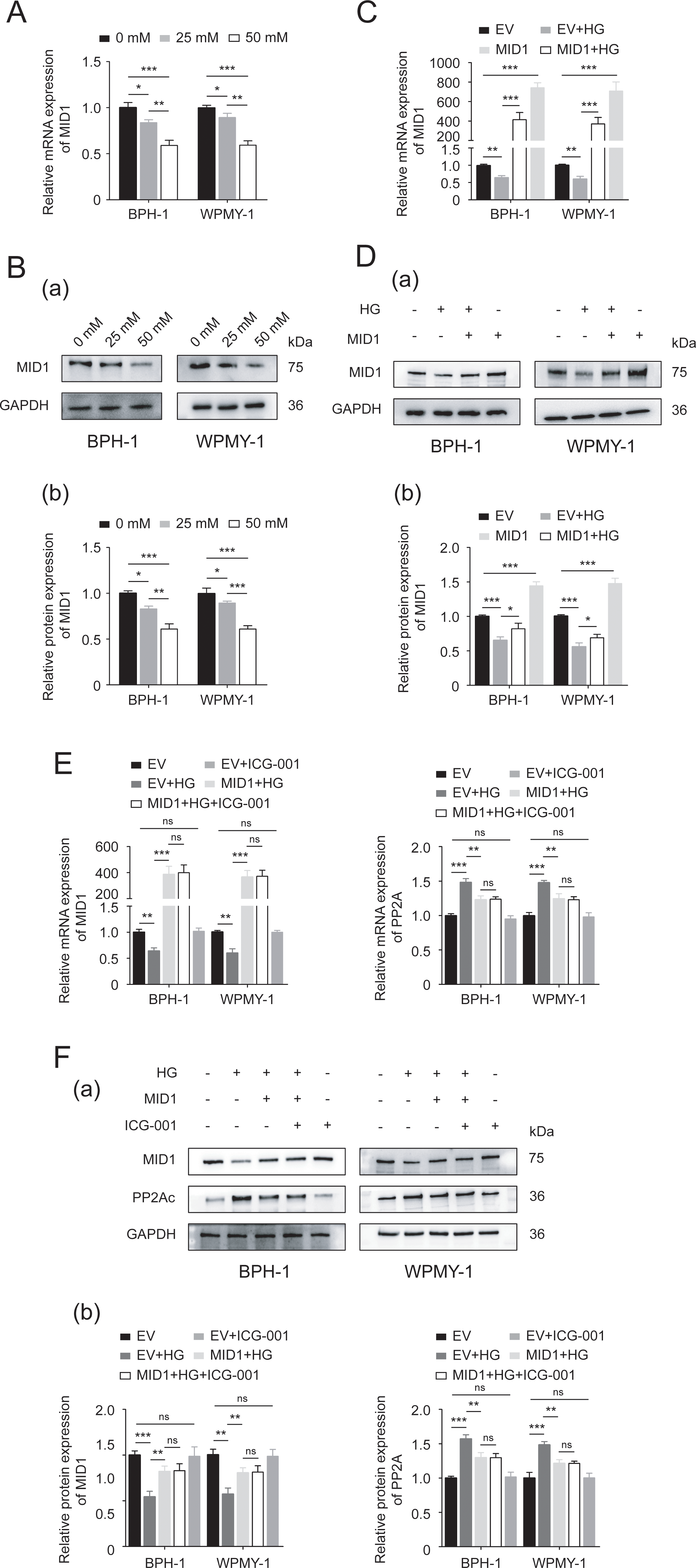

Supplement: Supplementary Figure 2 — The expression of MID1 and PP2A levels in prostatic cells upon various experimental treatments. (A) The mRNA expression of MID1 in BPH-1 and WPMY-1 cells undergoing 0, 25 and 50 mM glucose treatment. (B) Immunoblot assay (a) and relative densitometric quantification (b) for MID1 expression in high glucose (HG)-treated two prostatic cell lines. (C, D) The expression of MID1 at transcriptional and translational levels in BPH-1 and WPMY-1 cells upon HG treatment (+)/(/) and MID1 plasmid/EV transfection shown by qPCR (C), as well as western blot (D (a)) and relative densitometric quantification (D (b)). (E, F) The mRNA and protein expression of MID1 and PP2A levels in prostatic cell lines treated with MID1 overexpression (+)/(-), high glucose (+)/(-) and ICG-001 (+)/(-). GAPDH is used as loading control. ns: p > 0.05; *p < 0.05; **p < 0.01; ***p < 0.001. [file Image2.tif]

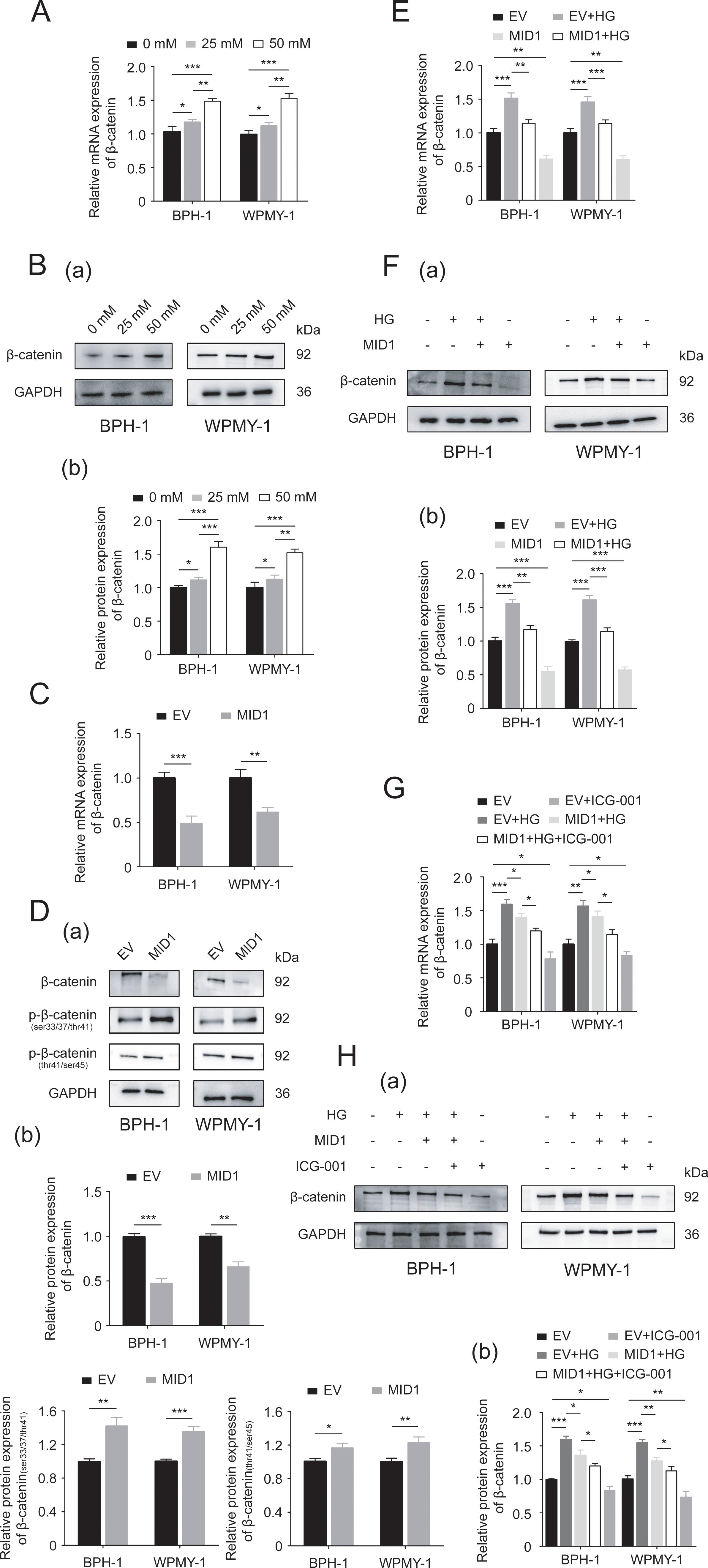

Supplement: Supplementary Figure 3 — The status of WNT/β-catenin signaling within two prostate cell lines upon various experimental treatments. (A) The mRNA and protein expression of β-catenin levels in prostatic cell lines (BPH-1 and WPMY-1) treated with increasing glucose concentrations (0, 25, 50 mM). (B) Immunoblot assay (a) and relative densitometric quantification (b) for expression of β-catenin in two prostatic cell lines following 0, 25, 50 mM glucose treatment. (C, D) The expression of β-catenin and phospho-β-catenin (ser33/37/thr41 & thr41/ser45) at transcriptional and translational levels in either MID1 plasmid- or empty vector (EV)- transposed prostate cell lines. (E, F) The mRNA and protein expression of β-catenin in BPH-1 and WPMY-1 cell lines upon MID1 overexpression (+)/(-) and high glucose treatment (+)/(-). (G, H) The expression of β-catenin at both mRNA and protein levels in two prostatic cell lines treated with MID1 overexpression (+)/(-), high glucose (+)/(-) and ICG-001 (+)/(-). GAPDH is used as loading control. *p < 0.05; **p < 0.01; ***p < 0.001. [file Image3.tif]

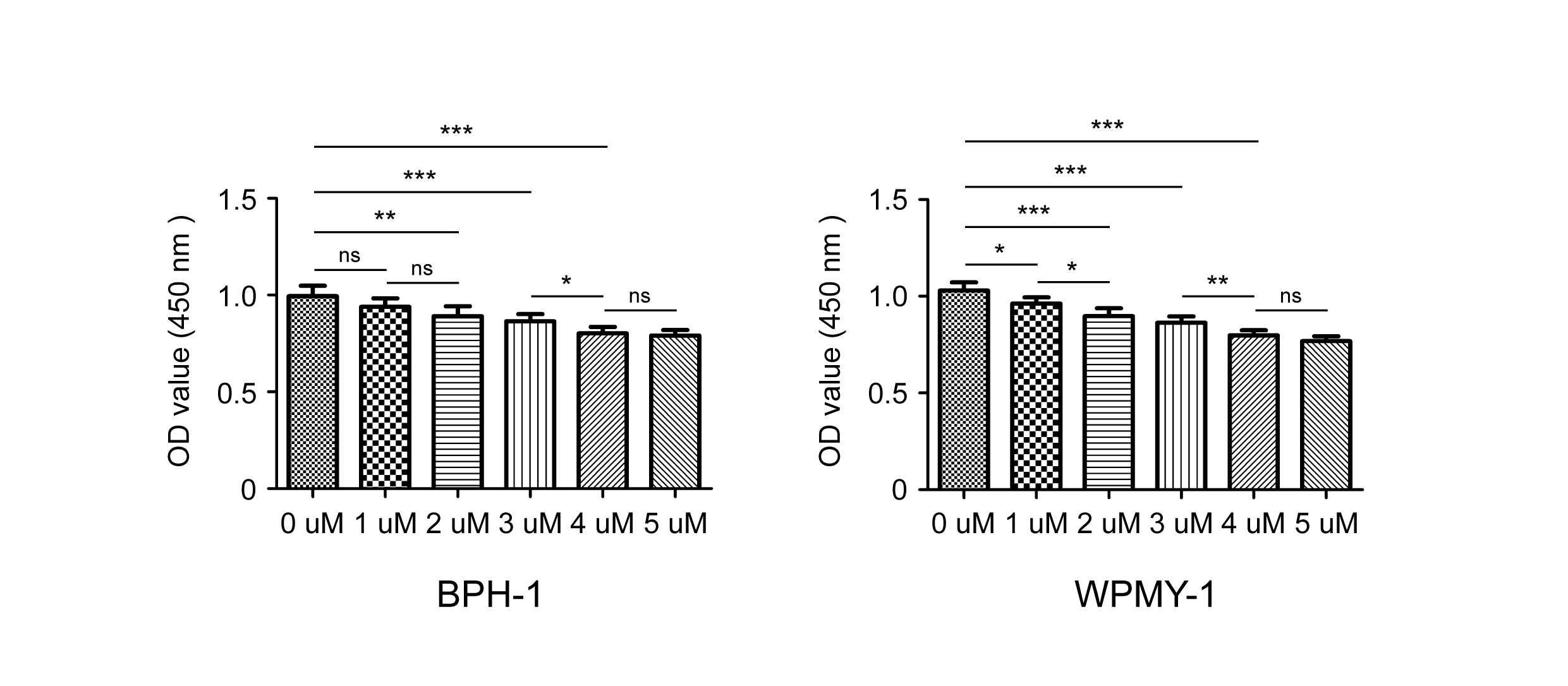

Supplement: Supplementary Figure 4 — Determination of experimental dose of ICG-001 for treatment of prostatic cell lines. Cell viability of BPH-1 and WPMY-1 cell lines upon treatment with increasing concentrations of ICG-001 for 48 h. ns: p > 0.05; *p < 0.05; **p < 0.01; ***p < 0.001. [file Image4.tif]
