# Supplementary material for: Midline-1 inhibited high glucose-induced epithelial-mesenchymal transition, fibrosis and inflammation through WNT/β-catenin signaling in benign prostatic hyperplasia
Source: Front Endocrinol (Lausanne). 2025 Mar 26;16:1543295. doi: 10.3389/fendo.2025.1543295 (PMC11978649; doi:10.3389/fendo.2025.1543295)
Supplement: Supplementary file 5 [file Table1.docx]

Table S1 Primer sequences of genes of interest for qRT-PCR

| gene (human) | forward primer (5’~3’) | reverse primer (5’~3’) |
| --- | --- | --- |
| MID1 | AGAGTGCGTGTAGCAACAG | CAGACAAATAGGGCAGGTCAG |
| PP2Ac | ATGGACGAGAAGTTGTTCAC | GACCACCATGTAGACAGAAG |
| IL-6 | TGGCTGCAGGACATGACAACT | ATCTGAGGTGCCCATGCTACA |
| IL-8 | TTTTGCCAAGGAGTGCTAAAGA | AACCCTCTGCACCCAGTTTTC |
| TNF-α | GCGGGAAATATGACAGCTAAGG | TGCTTGTCTGGAACAACTGC |
| α-SMA | GGCATTCACGAGACCACCTAC | CGACATGACGTTGTTGGCATAC |
| collagen-I | GAGGGCCAAGACGAAGACATC | CAGATCACGTCATCGCACAAC |
| E-cad | CGAGAGCTACACGTTCACGG | GGGTGTCGAGGGAAAAATAGG |
| N-cad | TCAGGCGTCTGTAGAGGCTT | ATGCACATCCTTCGATAAGACTG |
| vimentin | GACGCCATCAACACCGAGTT | CTTTGTCGTTGGTTAGCTGGT |
| β-catenin | ACTAAACAGGAAGGGATGGAAGG | AGATGACGAAGAGCACAGATGG |
| GAPDH | ATGGAGAAGGCTGGGGCTC | AAGTTGTCATGGATGACCTTG |
