# Supplementary material for: Midline-1 inhibited high glucose-induced epithelial-mesenchymal transition, fibrosis and inflammation through WNT/β-catenin signaling in benign prostatic hyperplasia
Source: Front Endocrinol (Lausanne). 2025 Mar 26;16:1543295. doi: 10.3389/fendo.2025.1543295 (PMC11978649; doi:10.3389/fendo.2025.1543295)
Supplement: Supplementary file 6 [file Table2.docx]

Table S2 Primary antibodies for Western Blot and immunofluorescence

| Antigens | Species & Type | Dilution | Supplier |
| --- | --- | --- | --- |
| MID1 | Rabbit, polyclonal | 1:1000 (WB)  1:50 (IF)  1:50 (IHC) | Abclonal (A7291) |
| PP2Ac | Rabbit | 1:1000 (WB)  1:50 (IF)  1:50 (IHC) | Abclonal (A6702) |
| IL-6 | Rabbit, monoclonal | 1:1000 (WB) | Abclonal (A22222) |
| IL-8 | Rabbit, polyclonal | 1:1000 (WB) | Abclonal (A2541) |
| TNF-α | Rabbit, polyclonal | 1:1000 (WB) | Abclonal (A11534) |
| α-SMA | Rabbit, polyclonal | 1:1000 (WB) | Abclonal (A7248) |
| collagen-I | Rabbit, polyclonal | 1:500 (WB) | Abclonal (A5786) |
| E-cad | Rabbit, monoclonal | 1:1000 (WB) | Abclonal (A20798) |
| N-cad | Rabbit, monoclonal | 1:1000 (WB) | Abclonal (A19083) |
| vimentin | Rabbit, polyclonal | 1:1000 (WB) | Abclonal (A11952) |
| β-catenin | Rabbit, monoclonal | 1:1000 (WB) | Abclonal (A19657) |
| phospho-β-catenin  (Ser33/37/Thr41) | Rabbit, polyclonal | 1:1000 (WB) | Cell Signaling (#9561) |
| phospho-β-catenin  (Thr41/Ser45) | Rabbit, polyclonal | 1:1000 (WB) | Cell Signaling (#9565) |
| GAPDH | Rabbit, polyclonal | 1:1000 (WB) | Abclonal (AC027) |
