# Supplementary material for: Midline-1 inhibited high glucose-induced epithelial-mesenchymal transition, fibrosis and inflammation through WNT/β-catenin signaling in benign prostatic hyperplasia
Source: Front Endocrinol (Lausanne). 2025 Mar 26;16:1543295. doi: 10.3389/fendo.2025.1543295 (PMC11978649; doi:10.3389/fendo.2025.1543295)
Supplement: Supplementary file 7 [file Table3.docx]

Table S3 Secondary antibodies for Western Blot and immunofluorescence

| Antigens | Species | Dilution | Supplier |
| --- | --- | --- | --- |
| Anti-Rabbit-IgG (H+L)-HRP | Goat | 1:10000 (WB) | Sungene Biotech, China, Cat. #LK2001 |
| Anti-rabbit IgG (H+L), F (ab')2 fragment (Alexa Fluor® 488 Conjugate) | Goat | 1:50 (IF) | Cell Signaling Technology, USA, cat. no. 4412 |
| Hoechst 33342 (1 mg/ml) nucleic acid staining (DAPI) | - | 1:750 (IF) | Molecular Probes/Invitrogen, Carlsbad, CA, USA, cat. no. A11007 |
